# Association between prognostic nutritional index and mortality in critically ill patients with atrial fibrillation: a retrospective cohort study

**DOI:** 10.1186/s12872-026-06138-4

**Published:** 2026-06-20

**Authors:** Xiangjun Kong, Zongjun Hu, Xia Huang, Jianguo Chen, Li Chen, Lei Huang, Shunlin Tan

**Affiliations:** 1 Department of Cardiology, The Second People’s Hospital of Neijiang, Affiliated Neijiang Hospital of Southwest Medical University, Neijiang, 641000 Sichuan People’s Republic of China; 2 Department of Critical Care Medicine, Chongqing University Three Gorges Hospital, Wanzhou, Chongqing, 404100 People’s Republic of China; 3 Department of Cardiology, Ziyang Central Hospital, Ziyang Hospital of West China Hospital, Sichuan University, Ziyang, 641300 Sichuan People’s Republic of China

**Keywords:** Prognostic nutritional index, Atrial fibrillation, Critically ill patients, Mortality, Intensive care

## Abstract

**Background:**

The prognostic nutritional index (PNI), a composite marker derived from serum albumin and total lymphocyte count, has been associated with adverse outcomes in several cardiovascular and critical care settings. However, its prognostic value in critically ill patients with atrial fibrillation (AF) remains unclear. This study aimed to evaluate the association between PNI and mortality in this high-risk population.

**Methods:**

This retrospective cohort study included critically ill patients with AF from the Medical Information Mart for Intensive Care IV (MIMIC-IV, version 3.1). The primary outcome was 30-day all-cause mortality. Secondary outcomes included in-hospital mortality, 90- and 365-day mortality. Multivariable Cox proportional hazards and logistic regression models were used to assess the associations between PNI and outcomes. Kaplan-Meier survival analyses and restricted cubic spline (RCS) analyses were performed to further characterize these associations. The association between PNI and in-hospital mortality was further examined in an independent cohort from the eICU Collaborative Research Database (eICU-CRD).

**Results:**

A total of 3,007 critically ill patients with AF were included in the MIMIC-IV cohort, and 2,741 patients were included in the independent eICU-CRD cohort. In the primary model (Model 2), each 10-unit increase in PNI was associated with a lower risk of 30-day mortality (HR, 0.63; 95% CI, 0.57–0.70; *P* < 0.001), 90-day mortality (HR, 0.64; 95% CI, 0.58–0.70; *P* < 0.001), and 365-day mortality (HR, 0.68; 95% CI, 0.63–0.74; *P* < 0.001) in the MIMIC-IV cohort. Higher PNI was also associated with lower in-hospital mortality in the MIMIC-IV cohort (OR, 0.58; 95% CI, 0.50–0.66; *P* < 0.001). In the independent eICU-CRD cohort, higher PNI remained significantly associated with lower in-hospital mortality (OR, 0.66; 95% CI, 0.57–0.76; *P* < 0.001). Kaplan-Meier analyses showed progressively better survival across increasing PNI quartiles, whereas RCS analyses demonstrated a nonlinear inverse relationship between PNI and mortality.

**Conclusions:**

Lower PNI was independently associated with increased short- and long-term mortality in critically ill patients with AF. As a readily available laboratory-based index, PNI may provide simple adjunctive prognostic information in this high-risk population.

**Supplementary Information:**

The online version contains supplementary material available at https://doi.org/10.1186/s12872-026-06138-4.

## Introduction

Atrial fibrillation (AF) is one of the most common arrhythmias in critically ill patients. Pre-existing AF is present in approximately 10%–20% of this population [1, 2], whereas new-onset AF develops in about 5%–15% of patients during critical illness [3, 4]. Both pre-existing and new-onset AF are associated with prolonged hospitalization, increased thromboembolic risk, and higher short- and long-term mortality [5–7]. Importantly, the adverse prognostic impact of AF in critical illness may reflect not only the arrhythmia itself but also the broader physiological derangements associated with critical illness [8, 9].

The prognostic nutritional index (PNI), calculated from serum albumin and total lymphocyte count, was originally developed as an immunological and nutritional marker [10]. In critically ill patients, both serum albumin and total lymphocyte count can be affected by acute pathophysiological changes [11–13]. Consequently, PNI should not be considered solely as a marker of nutritional status; it may also reflect broader inflammatory and nutritional vulnerability and reduced physiological reserve [14]. Lower PNI has been associated with adverse outcomes in several cardiovascular conditions and critically ill populations [15–18]. However, critically ill patients with AF represent a common and high-risk ICU subgroup in whom systemic inflammation, hemodynamic stress, organ dysfunction, and impaired cardiovascular reserve frequently coexist [9]. Although the prognostic role of PNI has been explored in broader ICU populations, its association with short- and long-term mortality has not been specifically characterized in critically ill patients with AF.

Using the Medical Information Mart for Intensive Care IV (MIMIC-IV) database as the primary cohort, we conducted this retrospective cohort study to evaluate the association between PNI and short- and long-term mortality among critically ill patients with AF. We further examined the dose-response relationship and potential threshold effect, and assessed the association between PNI and in-hospital mortality in an independent eICU Collaborative Research Database (eICU-CRD) cohort.

## Methods

### Data source

The primary cohort was obtained from the Medical Information Mart for Intensive Care IV (MIMIC-IV, version 3.1), which contains detailed information on 94,458 ICU admissions at Beth Israel Deaconess Medical Center in Boston, Massachusetts, USA, between 2008 and 2022 [19]. The independent cohort was obtained from the eICU Collaborative Research Database (eICU-CRD, version 2.0), a multicenter database containing clinical data from 200,859 ICU admissions across 208 hospitals in the United States between 2014 and 2015 [20].

Because both databases contain de-identified patient data, the requirement for informed consent was waived by the relevant institutional review boards. Database access was obtained by Zongjun Hu after completion of the Collaborative Institutional Training Initiative (CITI) training (certification ID: 60604020). This study was conducted in accordance with the Declaration of Helsinki and reported in accordance with the Strengthening the Reporting of Observational Studies in Epidemiology (STROBE) statement, with the completed STROBE checklist provided as supplementary material.

## Study population

**Fig. 1:**
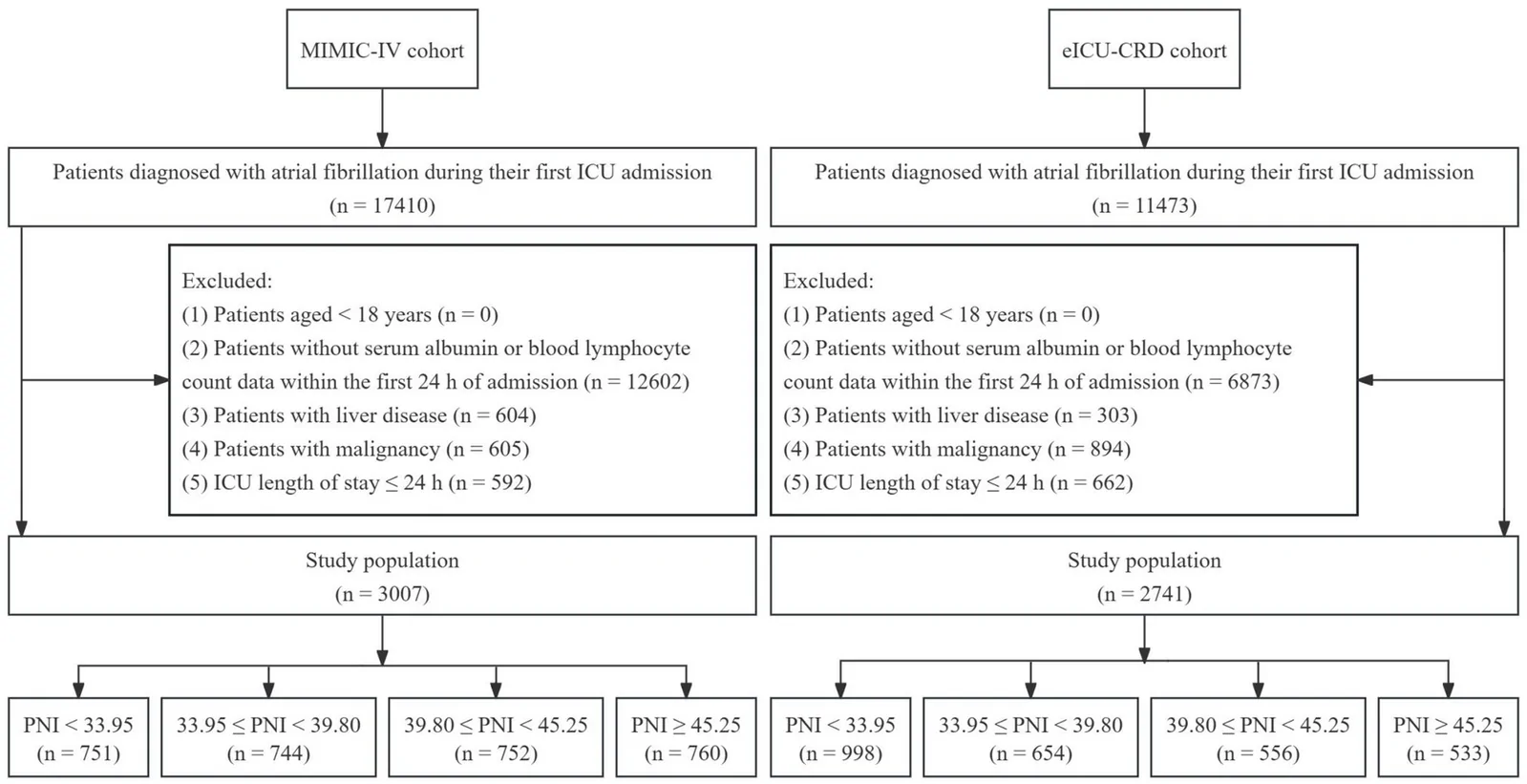
Flowchart of participant selection in the MIMIC-IV and eICU-CRD cohorts. PNI quartiles were were defined according to the MIMIC-IV cohort. Abbreviations: MIMIC-IV, Medical Information Mart for Intensive Care IV; eICU-CRD, eICU Collaborative Research Database; ICU, intensive care unit; PNI, prognostic nutritional index

Patients with AF during their first ICU admission were eligible for inclusion. The study population included both patients with pre-existing AF and those with new-onset AF (NOAF) occurring during the index ICU stay. In the MIMIC-IV database, AF was identified using ICD-9/10 diagnosis codes from the diagnoses_icd table. In the eICU-CRD database, AF was identified from the diagnosis table using the recorded diagnosis text (diagnosisstring). The detailed diagnostic criteria are provided in Supplementary Table S1. The exclusion criteria were as follows: (1) age < 18 years; (2) missing serum albumin, white blood cell count, or lymphocyte percentage data within the first 24 h after ICU admission; (3) liver disease; (4) malignancy; and (5) ICU length of stay ≤ 24 h. The detailed patient selection process is shown in Fig. 1.

### Data extraction

Clinical and demographic data were extracted from both databases using structured query language (SQL). Demographic variables included age and sex. Clinical parameters included comorbidities, severity scores, vital signs, laboratory values, and therapeutic interventions. Comorbidities were identified from diagnosis records. Vital signs and laboratory values were defined using the first recorded measurements within 24 h of ICU admission, and therapeutic interventions initiated during the same period were also recorded. The specific medications included in each treatment category are listed in Supplementary Table S2.

### Calculation of PNI

PNI was calculated based on the first recorded serum albumin, white blood cell count, and lymphocyte percentage values within the first 24 h after ICU admission. Total lymphocyte count (×10^9^/L) was calculated as white blood cell count (×10^9^/L) × lymphocyte percentage (%) / 100. PNI was then calculated as 10 × serum albumin (g/dL) + 5 × total lymphocyte count (×10^9^/L) [10].

### Endpoints

In the MIMIC-IV cohort, the primary endpoint was 30-day all-cause mortality, whereas the secondary endpoints were in-hospital mortality, 90-day mortality, and 365-day mortality. In the eICU-CRD cohort, only in-hospital mortality was assessed because long-term follow-up data were unavailable.

### Statistical analysis

Patients in the MIMIC-IV cohort were categorized into quartiles based on PNI (Q1–Q4). The quartile cutoffs derived from the MIMIC-IV cohort were then applied to classify patients in the eICU-CRD cohort into four corresponding groups (Groups 1–4). Continuous variables were presented as medians with interquartile ranges (IQRs) and compared using the Kruskal-Wallis test. Categorical variables were summarized as counts (percentages) and compared using the chi-square test or Fisher’s exact test, as appropriate. Missing values were minimal in the MIMIC-IV cohort (< 0.5% for all variables) and remained low in the eICU-CRD cohort (< 5% for all variables) (Supplementary Table S3). Missing covariate data were handled using multiple imputation by chained equations with the mice package in R [21], and estimates from 10 imputed datasets were combined using Rubin’s rules [22].

Associations between PNI and time-to-event outcomes (30-day, 90-day, and 365-day all-cause mortality) were assessed using multivariable Cox proportional hazards models, whereas the association with in-hospital mortality was evaluated using multivariable logistic regression. The proportional hazards assumption for PNI was examined using scaled Schoenfeld residuals in the adjusted Cox models for 30-day, 90-day, and 365-day mortality. No statistically significant violation was observed for PNI across the three endpoints, as shown in Supplementary Table S11 and Supplementary Figure S1. Effect estimates were reported as hazard ratios (HRs) or odds ratios (ORs) with 95% confidence intervals (CIs). PNI was modeled both as a categorical variable (Q1–Q4 in MIMIC-IV and Groups 1–4 in eICU-CRD) and as a continuous variable (per 10-unit increase). Kaplan-Meier survival curves were constructed across PNI groups and compared using the log-rank test. Covariates were selected on the basis of clinical relevance, a > 10% change-in-estimate criterion, and assessment of variance inflation factors (VIFs) to minimize multicollinearity (Supplementary Table S4). Because acute severity scores and early ICU interventions may lie along the pathway between PNI and subsequent outcomes, or may represent downstream manifestations of early illness severity, Model 2 (excluding SAPS II and ICU interventions) was prespecified as the primary model, whereas Model 3 (including these variables) was treated as a sensitivity analysis. Four models were constructed: a crude model (unadjusted); Model 1, adjusted for age and sex; Model 2, adjusted for age, sex, hypertension, cerebrovascular disease, diabetes, chronic kidney disease (CKD), congestive heart failure (CHF), heart rate, respiratory rate, systolic blood pressure (SBP), hemoglobin, creatinine, and bicarbonate; and Model 3, further adjusted for the Simplified Acute Physiology Score II (SAPS II), vasopressor use, mechanical ventilation, and renal replacement therapy (RRT).

RCS analyses were fitted using four knots at the 5th, 35th, 65th, and 95th percentiles to characterize the dose-response relationship between continuous PNI and the mortality endpoints (30-day, 90-day, and 365-day) and to test for potential nonlinearity. For the primary outcome (30-day mortality), if a nonlinear association was identified, a two-segment piecewise Cox proportional hazards model was further fitted to evaluate the threshold effect. The inflection point was identified using a recursive algorithm that maximized the model likelihood, and the fit of the piecewise model was compared with that of the standard linear model using a likelihood ratio test.

Subgroup analyses were performed to evaluate the consistency of the association across age (< 65 vs. ≥65 years), sex, diabetes, CKD, CHF, and AF type (pre-existing AF vs. new-onset AF [NOAF]). Furthermore, to distinguish pre-existing AF from NOAF, we identified NOAF in the MIMIC-IV cohort using bedside rhythm records (itemid 220048). NOAF was operationally defined as the first recorded episode of AF during the ICU stay that was preceded by a documented period of sinus rhythm. Stratified analyses were conducted using the primary adjusted model (Model 2), with the corresponding stratification variable excluded as a covariate where appropriate. P values for interaction were derived from likelihood ratio tests to assess potential effect modification across subgroups.

To further describe the prognostic information provided by PNI, the C-statistic for 30-day mortality was compared between PNI and its individual components, including serum albumin and total lymphocyte count. Continuous net reclassification improvement (NRI) and integrated discrimination improvement (IDI) were calculated as supplementary measures of reclassification and discrimination improvement [23]. These analyses were used to examine whether the composite index provided additional prognostic information beyond its individual components. Furthermore, PNI was incorporated into base models containing established severity scores, including SAPS II and APACHE II, to examine whether it provided additional prognostic information beyond conventional ICU severity assessment. Differences in these supplementary measures were summarized using the C-statistic, continuous NRI, and IDI. As an additional exploratory analysis, the C-statistic was also compared between SOFA alone and SOFA plus PNI.

Several sensitivity analyses were performed for 30-day mortality in the MIMIC-IV cohort. First, analyses were repeated after excluding patients with CHF, CAD, or valvular heart disease, as well as those who died within 48 h of ICU admission, to examine the robustness of the primary association and to partly address the possibility that a low PNI merely reflected imminent death. Second, to evaluate the robustness of adjustment for chronic disease burden, individual comorbidities were replaced with the Charlson Comorbidity Index (CCI). Finally, Model 3 was used to assess the independent association between PNI and mortality after further adjustment for acute illness severity (SAPS II) and ICU interventions. An additional sensitivity analysis was performed by replacing SAPS II with SOFA score, while retaining adjustment for ICU interventions, to assess the robustness of the association after accounting for organ dysfunction severity.

To further evaluate the robustness of the primary association to potential unmeasured confounding, E-values were calculated for the point estimate and for the confidence limit closest to the null for the primary endpoint. The E-value quantifies the minimum strength of association that an unmeasured confounder would need to have with both the exposure and the outcome, conditional on the measured covariates, to fully explain away the observed association [24].

Bootstrap resampling with 1,000 iterations was used to assess the internal stability of the primary association in the MIMIC-IV cohort, and bootstrap-based mean estimates with percentile-based 95% confidence intervals were calculated. The eICU-CRD cohort was used to further examine the association between PNI and in-hospital mortality in an independent cohort.

All analyses were performed using R Statistical Software (version 4.2.2; R Foundation for Statistical Computing, Vienna, Austria). A two-sided P-value < 0.05 was considered statistically significant.

## Results

### Study population and baseline characteristics

A total of 3,007 eligible patients from the MIMIC-IV cohort were included in the primary analysis (median age, 77.8 years; 56.0% were male). The overall in-hospital, 30-day, 90-day, and 365-day mortality rates in this cohort were 19.1%, 24.1%, 31.1%, and 40.9%, respectively. The independent eICU-CRD cohort comprised 2,741 patients (median age, 76.0 years) with an overall in-hospital mortality rate of 15.2%.

In the MIMIC-IV cohort, patients were categorized into four quartiles based on PNI (Q1–Q4). Detailed baseline characteristics and crude outcomes across PNI categories for the MIMIC-IV and eICU-CRD cohorts are summarized in Table 1 and Supplementary Table S5, respectively. In the MIMIC-IV cohort, a clear inverse trend was observed across PNI quartiles for all mortality endpoints (all *P* < 0.001). Specifically, compared with those in the highest quartile (Q4), patients in the lowest quartile (Q1) exhibited substantially higher mortality across all assessed time points, including in-hospital mortality (29.3% vs. 14.2%), 30-day mortality (35.6% vs. 17.2%), 90-day mortality (44.2% vs. 22.2%), and 365-day mortality (53.9% vs. 30.4%). A consistent and significant trend for in-hospital mortality was observed in the eICU-CRD cohort (22.1% in Group 1 vs. 8.4% in Group 4, *P* < 0.001).

**Table 1:** Baseline characteristics according to PNI quartiles in the MIMIC-IV cohort

Variables	Total (*n* = 3007)	Q1 (*n* = 751)	Q2 (*n* = 744)	Q3 (*n* = 752)	Q4 (*n* = 760)	*P* value
Demographics						
Age, years	77.8 (68.3–85.4)	77.9 (68.8–85.0)	79.8 (70.7–86.1)	78.4 (69.3–85.7)	75.4 (66.2–84.2)	< 0.001
Male, n (%)	1685 (56.0)	408 (54.3)	446 (59.9)	391 (52.0)	440 (57.9)	0.009
Comorbidities, n (%)						
Hypertension	2329 (77.5)	540 (71.9)	587 (78.9)	598 (79.5)	604 (79.5)	< 0.001
Cerebrovascular disease	801 (26.6)	125 (16.6)	145 (19.5)	218 (29.0)	313 (41.2)	< 0.001
COPD	844 (28.1)	226 (30.1)	237 (31.9)	238 (31.6)	143 (18.8)	< 0.001
Diabetes	1047 (34.8)	280 (37.3)	274 (36.8)	272 (36.2)	221 (29.1)	0.002
CKD	939 (31.2)	251 (33.4)	265 (35.6)	251 (33.4)	172 (22.6)	< 0.001
CAD	1305 (43.4)	329 (43.8)	337 (45.3)	321 (42.7)	318 (41.8)	0.563
CHF	1575 (52.4)	408 (54.3)	452 (60.8)	415 (55.2)	300 (39.5)	< 0.001
Valvular heart disease	661 (22.0)	163 (21.7)	193 (25.9)	167 (22.2)	138 (18.2)	0.004
Severity scores						
CCI	6.0 (5.0–8.0)	6.0 (5.0–8.0)	7.0 (5.0–8.0)	7.0 (5.0–8.0)	6.0 (5.0–8.0)	< 0.001
SAPS II	40.0 (33.0–50.0)	46.0 (37.0–56.0)	42.0 (35.0–51.0)	38.0 (32.0–46.0)	35.0 (29.0–44.0)	< 0.001
APACHE II	19.0 (15.0–25.0)	22.0 (18.0–27.0)	20.0 (16.0–26.0)	18.0 (15.0–24.0)	16.0 (12.0–21.0)	< 0.001
Vital signs						
Temperature, °C	36.7 (36.4–37.0)	36.7 (36.4–37.1)	36.7 (36.4–37.1)	36.7 (36.4–37.0)	36.7 (36.4–36.9)	0.617
Heart rate, beats/min	87.0 (74.0–104.0)	92.0 (79.0–110.0)	87.5 (74.0–103.0)	85.0 (72.0–103.0)	83.0 (70.8–98.0)	< 0.001
Respiratory rate, breaths/min	20.0 (16.0–23.0)	20.0 (17.0–25.0)	20.0 (16.0–24.0)	19.0 (16.0–23.0)	19.0 (16.0–22.0)	< 0.001
SBP, mmHg	123.0 (106.0–141.0)	116.0 (101.0–133.0)	120.0 (105.8–138.0)	125.0 (108.0–143.0)	130.0 (114.0–147.0)	< 0.001
DBP, mmHg	68.0 (56.0–81.0)	64.0 (53.0–76.0)	66.0 (57.0–78.0)	70.0 (58.0–82.0)	73.0 (60.0–86.0)	< 0.001
SpO₂, %	97.0 (95.0–100.0)	97.0 (94.0–100.0)	97.0 (94.0–100.0)	97.0 (95.0–100.0)	97.0 (95.0–99.0)	0.020
Laboratory tests						
WBC, ×10⁹/L	11.3 (8.1–16.0)	12.4 (8.4–18.1)	11.6 (8.1–16.6)	10.9 (7.8–14.6)	10.6 (8.2–14.7)	< 0.001
Hemoglobin, g/dL	11.5 (9.8–13.3)	10.2 (8.8–11.9)	11.0 (9.4–12.6)	11.8 (10.2–13.1)	13.1 (11.7–14.4)	< 0.001
Platelet count, ×10⁹/L	205.0 (156.0–269.0)	200.0 (147.5–282.0)	198.0 (142.0–266.0)	205.0 (161.0–261.0)	216.0 (171.0–269.0)	< 0.001
Creatinine, mg/dL	1.2 (0.9–1.9)	1.4 (0.9–2.3)	1.3 (0.9–2.1)	1.2 (0.9–1.8)	1.1 (0.9–1.5)	< 0.001
Blood urea nitrogen, mg/dL	27.0 (18.0–44.0)	34.0 (20.0–54.0)	30.0 (20.0–50.0)	26.0 (18.0–39.0)	21.0 (16.0–30.0)	< 0.001
Bicarbonate, mmol/L	22.0 (19.0–25.0)	21.0 (18.0–25.0)	22.0 (19.0–25.0)	23.0 (20.0–25.0)	23.0 (20.0–25.0)	< 0.001
Sodium, mmol/L	138.0 (135.0–141.0)	138.0 (135.0–142.0)	138.0 (135.0–141.0)	138.0 (135.0–141.0)	139.0 (136.0–142.0)	< 0.001
Chloride, mmol/L	102.0 (98.0–106.0)	103.0 (98.0–108.0)	102.0 (97.0–107.0)	102.0 (98.0–105.0)	102.0 (99.0–105.0)	< 0.001
Potassium, mmol/L	4.3 (3.9–4.9)	4.2 (3.8–4.8)	4.4 (3.9–5.0)	4.4 (3.9–4.9)	4.2 (3.8–4.7)	< 0.001
Treatments, n (%)						
Beta-blocker use	1285 (42.7)	263 (35.0)	282 (37.9)	333 (44.3)	407 (53.6)	< 0.001
Anticoagulant use	1960 (65.2)	541 (72.0)	486 (65.3)	473 (62.9)	460 (60.5)	< 0.001
Diuretic use	998 (33.2)	221 (29.4)	293 (39.4)	273 (36.3)	211 (27.8)	< 0.001
Vasopressor use	836 (27.8)	320 (42.6)	223 (30.0)	153 (20.3)	140 (18.4)	< 0.001
Inotropic drug use	248 (8.2)	60 (8.0)	65 (8.7)	74 (9.8)	49 (6.4)	0.108
Mechanical ventilation	1236 (41.1)	385 (51.3)	326 (43.8)	264 (35.1)	261 (34.3)	< 0.001
RRT	139 (4.6)	54 (7.2)	36 (4.8)	28 (3.7)	21 (2.8)	< 0.001
Outcomes, n (%)						
In-hospital mortality	574 (19.1)	220 (29.3)	137 (18.4)	109 (14.5)	108 (14.2)	< 0.001
30-day mortality	724 (24.1)	267 (35.6)	181 (24.3)	145 (19.3)	131 (17.2)	< 0.001
90-day mortality	934 (31.1)	332 (44.2)	251 (33.7)	182 (24.2)	169 (22.2)	< 0.001
365-day mortality	1230 (40.9)	405 (53.9)	333 (44.8)	261 (34.7)	231 (30.4)	< 0.001

Data are presented as median (interquartile range [IQR]) or n (%), as appropriate. P values were calculated using the Kruskal-Wallis test for continuous variables and the chi-square test or Fisher’s exact test, as appropriate, for categorical variables. PNI quartiles were defined as follows: Q1, PNI < 33.95; Q2, 33.95 ≤ PNI < 39.80; Q3, 39.80 ≤ PNI < 45.25; and Q4, PNI ≥ 45.25

*Abbreviations PNI* prognostic nutritional index, *COPD* chronic obstructive pulmonary disease, *CKD* chronic kidney disease, *CAD* coronary artery disease, *CHF* congestive heart failure, *CCI* Charlson Comorbidity Index, *SAPS II* Simplified Acute Physiology Score II, *APACHE II* Acute Physiology and Chronic Health Evaluation II, *SBP* systolic blood pressure, *DBP* diastolic blood pressure, *SpO₂* peripheral oxygen saturation, *WBC* white blood cell count, *RRT* renal replacement therapy, *IQR* interquartile range

Across both cohorts, lower PNI categories had higher illness-severity scores and a greater burden of comorbidities. Patients in the lowest PNI group (Q1) had a higher prevalence of CHF and CKD, and markedly higher median severity scores and CCI values (all *P* < 0.001). For instance, the median SAPS II score in the MIMIC-IV cohort was 46.0 in Q1, compared with 35.0 in Q4 (*P* < 0.001). These patients more frequently required intensive clinical interventions, including vasopressor use, mechanical ventilation, and RRT.

### Association between PNI and mortality in the MIMIC-IV cohort

Multivariable regression analyses showed that higher PNI was consistently associated with lower mortality across all endpoints (Table 2). For the primary endpoint (30-day mortality), each 10-unit increase in PNI was associated with a lower risk of 30-day mortality (Model 2, HR, 0.63; 95% CI, 0.57–0.70; *P* < 0.001). When PNI was analyzed in quartiles, a significant dose-response relationship was observed; compared with Q1, the adjusted HRs for Q2, Q3, and Q4 were 0.63 (95% CI, 0.52–0.76), 0.50 (95% CI, 0.40–0.62), and 0.44 (95% CI, 0.35–0.56), respectively (*P* for trend < 0.001; Table 2).

**Table 2:** Associations between prognostic nutritional index and 30-day, 90-day, and 365-day mortality in the MIMIC-IV cohort

PNI	Crude model		Model 1		Model 2		Model 3	
	HR (95% CI)	*P* value	HR (95% CI)	*P* value	HR (95% CI)	*P* value	HR (95% CI)	*P* value
30-day mortality								
Per 10-unit increase	0.65 (0.59–0.71)	< 0.001	0.64 (0.59–0.71)	< 0.001	0.63 (0.57–0.70)	< 0.001	0.75 (0.68–0.83)	< 0.001
Q1	1.00 (Ref)		1.00 (Ref)		1.00 (Ref)		1.00 (Ref)	
Q2	0.63 (0.52–0.77)	< 0.001	0.60 (0.50–0.73)	< 0.001	0.63 (0.52–0.76)	< 0.001	0.70 (0.58–0.85)	< 0.001
Q3	0.49 (0.40–0.61)	< 0.001	0.48 (0.39–0.59)	< 0.001	0.50 (0.40–0.62)	< 0.001	0.66 (0.53–0.82)	< 0.001
Q4	0.44 (0.36–0.54)	< 0.001	0.46 (0.37–0.56)	< 0.001	0.44 (0.35–0.56)	< 0.001	0.60 (0.47–0.76)	< 0.001
P for trend		< 0.001		< 0.001		< 0.001		< 0.001
90-day mortality								
Per 10-unit increase	0.64 (0.59–0.69)	< 0.001	0.63 (0.58–0.69)	< 0.001	0.64 (0.58–0.70)	< 0.001	0.74 (0.68–0.82)	< 0.001
Q1	1.00 (Ref)		1.00 (Ref)		1.00 (Ref)		1.00 (Ref)	
Q2	0.69 (0.59–0.82)	< 0.001	0.65 (0.55–0.77)	< 0.001	0.68 (0.58–0.81)	< 0.001	0.76 (0.64–0.90)	0.001
Q3	0.48 (0.40–0.58)	< 0.001	0.46 (0.39–0.55)	< 0.001	0.49 (0.41–0.59)	< 0.001	0.63 (0.52–0.76)	< 0.001
Q4	0.44 (0.36–0.53)	< 0.001	0.45 (0.38–0.55)	< 0.001	0.47 (0.38–0.57)	< 0.001	0.61 (0.49–0.75)	< 0.001
P for trend		< 0.001		< 0.001		< 0.001		< 0.001
365-day mortality								
Per 10-unit increase	0.67 (0.63–0.72)	< 0.001	0.67 (0.62–0.72)	< 0.001	0.68 (0.63–0.74)	< 0.001	0.77 (0.71–0.84)	< 0.001
Q1	1.00 (Ref)		1.00 (Ref)		1.00 (Ref)		1.00 (Ref)	
Q2	0.74 (0.64–0.85)	< 0.001	0.69 (0.60–0.80)	< 0.001	0.72 (0.62–0.83)	< 0.001	0.78 (0.67–0.91)	0.001
Q3	0.54 (0.46–0.63)	< 0.001	0.51 (0.44–0.60)	< 0.001	0.55 (0.46–0.64)	< 0.001	0.66 (0.56–0.78)	< 0.001
Q4	0.46 (0.39–0.54)	< 0.001	0.48 (0.41–0.56)	< 0.001	0.52 (0.43–0.62)	< 0.001	0.64 (0.53–0.77)	< 0.001
P for trend		< 0.001		< 0.001		< 0.001		< 0.001

PNI quartiles were defined as follows: Q1, PNI < 33.95; Q2, 33.95 ≤ PNI < 39.80; Q3, 39.80 ≤ PNI < 45.25; and Q4, PNI ≥ 45.25

The crude model was unadjusted

Model 1 was adjusted for age and sex

Model 2 (primary model) was adjusted for age, sex, hypertension, cerebrovascular disease, diabetes, CKD, CHF, heart rate, respiratory rate, SBP, hemoglobin, creatinine, and bicarbonate

Model 3 (sensitivity model) was additionally adjusted for SAPS II, vasopressor use, mechanical ventilation, and RRT

*Abbreviations* *PNI* prognostic nutritional index, *HR* hazard ratio, *CI* confidence interval, *CKD* chronic kidney disease, *CHF* congestive heart failure, *SBP* systolic blood pressure, *SAPS II* Simplified Acute Physiology Score II, *RRT* renal replacement therapy

Regarding the secondary endpoints, each 10-unit increase in PNI was similarly associated with a lower risk of 90-day mortality (HR, 0.64; 95% CI, 0.58–0.70) and 365-day mortality (HR, 0.68; 95% CI, 0.63–0.74; both *P* < 0.001). Multivariable logistic regression also showed a significant association between higher PNI and lower in-hospital mortality (OR, 0.58; 95% CI, 0.50–0.66; *P* < 0.001).

In the sensitivity model (Model 3), which additionally included the acute severity score (SAPS II) and early ICU interventions, PNI remained significantly associated with 30-day mortality (HR, 0.76; 95% CI, 0.69–0.84; *P* < 0.001) and in-hospital mortality (OR, 0.71; 95% CI, 0.61–0.81; *P* < 0.001).

**Fig. 2:**
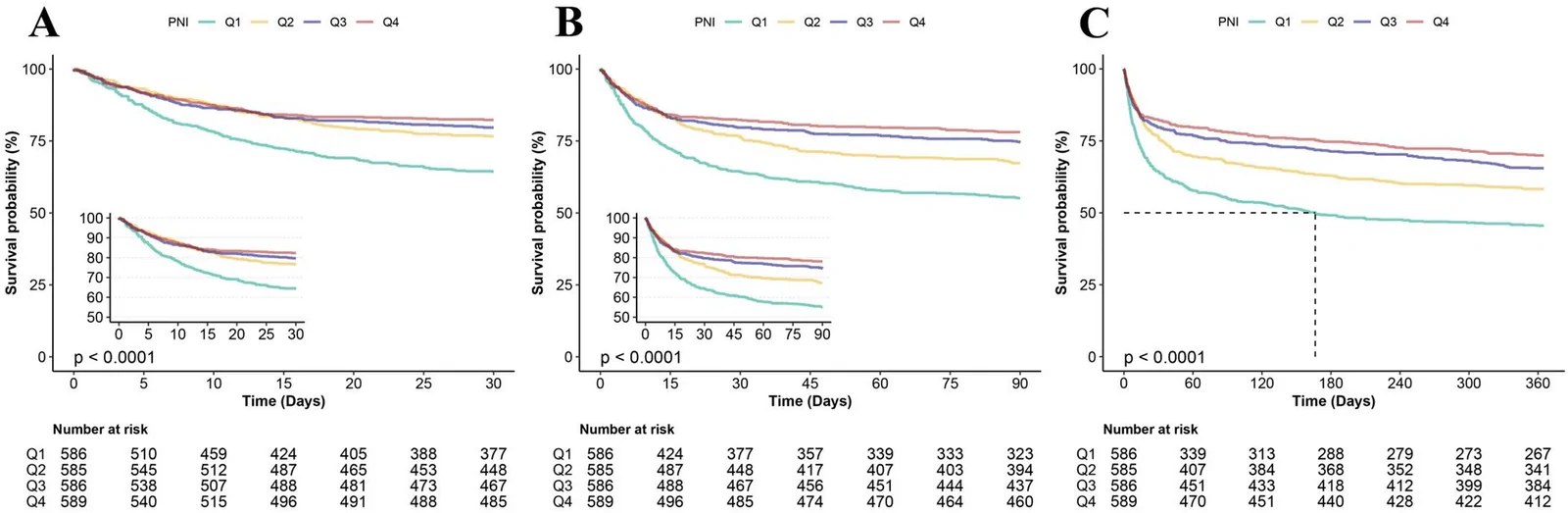
Kaplan–Meier survival curves for all-cause mortality according to PNI quartiles in the MIMIC-IV cohort. **A** 30-day all-cause mortality; (**B**) 90-day all-cause mortality; and (**C**) 365-day all-cause mortality. PNI quartiles were defined as follows: Q1, PNI < 33.95; Q2, 33.95 ≤ PNI < 39.80; Q3, 39.80 ≤ PNI < 45.25; and Q4, PNI ≥ 45.25. Survival differences across PNIquartiles were compared using the log-rank test (all *p* < 0.0001). The dashed line in panel C represents the 50% survival probability. Abbreviations: PNI, prognostic nutritional index

**Fig. 3:**
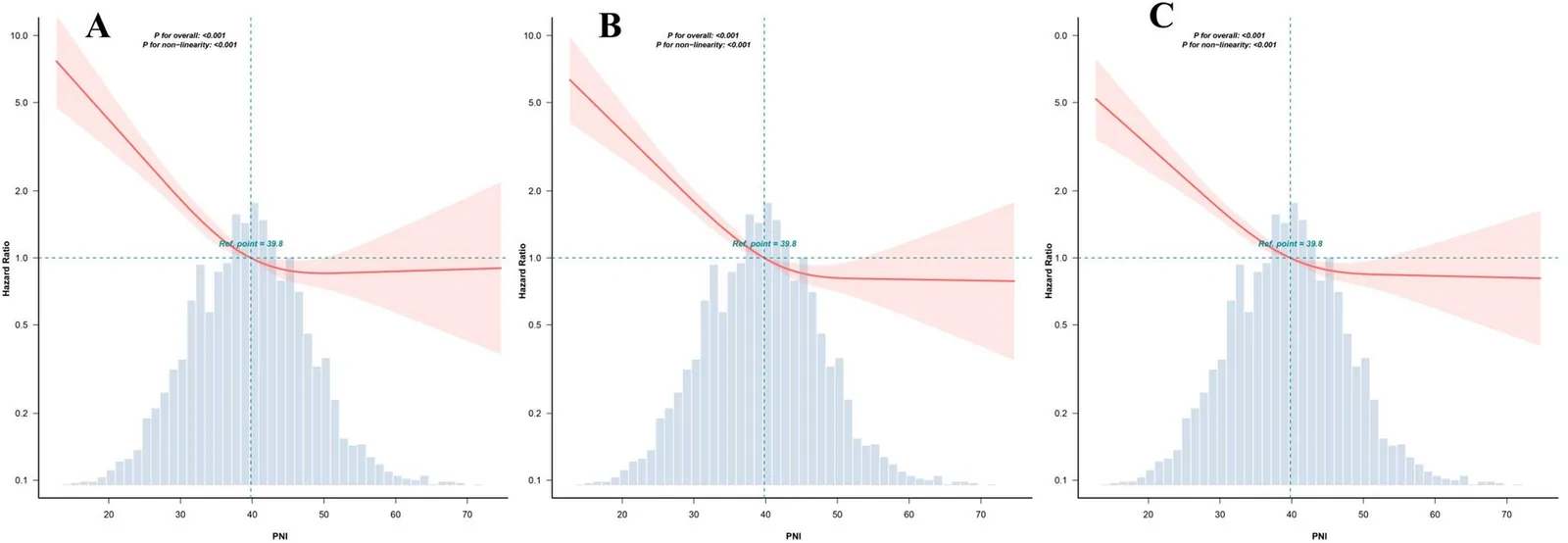
Restricted cubic spline analyses of the association between PNI and all-cause mortality in the MIMIC-IV cohort. Solid lines and shaded areas represent the predicted hazard ratios and 95% confidence intervals, respectively. Adjusted for age, sex, hypertension, cerebrovascular disease, diabetes, CKD, CHF, heart rate, respiratory rate, SBP, hemoglobin, creatinine, and bicarbonate. **A** 30-day all-cause mortality; (**B**) 90-day all-cause mortality; (**C**) 365-day all-cause mortality. Abbreviations: PNI, prognostic nutritional index; CKD, chronic kidney disease; CHF, congestive heart failure; SBP, systolic blood pressure

Kaplan-Meier survival curves showed clear separation across PNI quartiles, with progressively lower survival probabilities among patients with lower PNI values across the 30-day, 90-day, and 365-day follow-up periods (all log-rank *P* < 0.0001; Fig. 2). Restricted cubic spline (RCS) analyses showed significant nonlinear inverse relationships between PNI and all mortality endpoints (all *P* for nonlinearity < 0.001; Fig. 3). In the threshold analysis for the primary 30-day endpoint, an inflection point was identified at a PNI value of 39.82 (Table 3). Below this threshold, each 1-unit increase in PNI was associated with a 7% lower risk of 30-day mortality (HR, 0.93; 95% CI, 0.92–0.95; *P* < 0.001), whereas the association plateaued above this value (HR, 0.98; 95% CI, 0.96–1.01; *P* = 0.209), supporting the presence of a threshold effect (*P* for likelihood ratio test < 0.001).

**Table 3:** Threshold effect analysis of the association of PNI with 30-day mortality in the MIMIC-IV cohort

PNI range	Adjusted HR	95% CI	*P* value
PNI < 39.82	0.93	(0.92–0.95)	< 0.001
PNI ≥ 39.82	0.98	(0.96–1.01)	0.209
P for likelihood ratio test			< 0.001

The model was adjusted for age, sex, hypertension, cerebrovascular disease, diabetes, CKD, CHF, heart rate, respiratory rate, SBP, hemoglobin, creatinine, and bicarbonate

The HR represents the risk associated with each 1-unit increase in PNI

*Abbreviations* *PNI* prognostic nutritional index, *HR* hazard ratio, *CI* confidence interval, *CKD* chronic kidney disease, *CHF* congestive heart failure, *SBP* systolic blood pressure

**Fig. 4:**
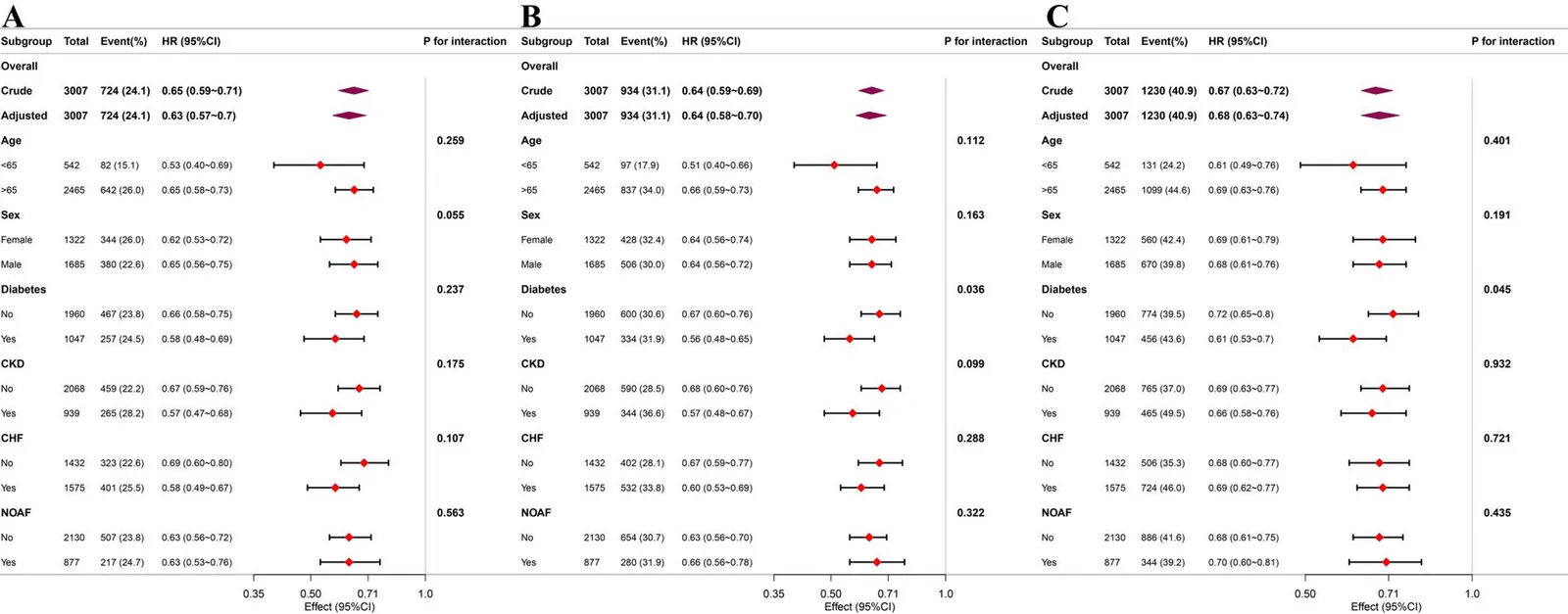
Subgroup analyses for the association between PNI (per 10-unit increase) and all-cause mortality in the MIMIC-IV cohort. Hazard ratios (HRs) and 95% confidence intervals (CIs) for each 10-unit increase in PNI are shown. Models were adjusted for age, sex, hypertension, cerebrovascular disease, diabetes, CKD, CHF, heart rate, respiratory rate, SBP, hemoglobin, creatinine, and bicarbonate (except for the variable used for stratification). *P* for interaction was used to assess potential effect modification across prespecified subgroups. **A** 30-day all-cause mortality; (**B**) 90-day all-cause mortality; (**C**) 365-day all-cause mortality. Abbreviations: PNI, prognostic nutritional index; HR, hazard ratio; CI, confidence interval; CKD, chronic kidney disease; CHF, congestive heart failure; NOAF, new-onset atrial fibrillation; SBP, systolic blood pressure

For the primary outcome of 30-day mortality, subgroup analyses showed generally consistent inverse associations across predefined strata, with no significant interactions observed (Fig. 4). For longer-term outcomes, however, significant interactions with diabetes status were identified. Specifically, for 90-day mortality, the HR was 0.56 (95% CI, 0.48–0.65) in patients with diabetes compared with 0.67 (95% CI, 0.60–0.76) in those without diabetes (*P* for interaction = 0.036). A similar pattern was observed for 365-day mortality (*P* for interaction = 0.045), suggesting that the inverse association between PNI and mortality may be more pronounced in patients with diabetes. No significant interactions were observed for age, sex, CKD, CHF, or AF type.

### Additional prognostic information provided by PNI

The discrimination performance of PNI for 30-day mortality was further compared with that of its individual components (Supplementary Table S6). The C-statistic of PNI was 0.612 (95% CI, 0.588–0.636), which was numerically higher than that of total lymphocyte count (0.591, 95% CI, 0.567–0.615; *P* = 0.080) and albumin (0.591, 95% CI, 0.566–0.616; *P* < 0.001). Although the absolute difference in C-statistic was modest, PNI showed statistically significant net reclassification gains compared with both individual components, with a continuous NRI of 0.115 (95% CI, 0.056–0.170; *P* < 0.001) versus total lymphocyte count and 0.120 (95% CI, 0.042–0.165; *P* = 0.02) versus albumin. Corresponding IDI values were 0.019 (*P* < 0.001) and 0.007 (*P* = 0.008), respectively.

The additional prognostic information provided by PNI beyond established severity scores was also evaluated (Supplementary Table S7). When added to SAPS II, the C-statistic changed from 0.711 to 0.717 (*P* = 0.094), with a continuous NRI of 0.095 (95% CI, 0.052–0.138; *P* < 0.001) and an IDI of 0.007 (95% CI, 0.002–0.017; *P* < 0.001). When added to APACHE II, the C-statistic changed from 0.687 to 0.698 (*P* = 0.015), with a continuous NRI of 0.105 (95% CI, 0.060–0.153; *P* < 0.001) and an IDI of 0.009 (95% CI, 0.003–0.020; *P* < 0.001). When PNI was added to SOFA, the C-statistic changed minimally from 0.666 (95% CI, 0.647–0.684) to 0.670 (95% CI, 0.651–0.689), and this change was not statistically significant (*P* = 0.305).

Sensitivity analyses and independent cohort analysis.

The inverse association between PNI and 30-day mortality remained significant in multiple sensitivity analyses (Supplementary Table S8). After excluding patients with CHF, CAD, valvular heart disease, or death within 48 h of ICU admission, each 10-unit increase in PNI remained significantly associated with lower 30-day mortality (adjusted HRs: 0.60–0.69; all *P* < 0.001). The association remained significant after replacing individual comorbidities with the CCI (HR, 0.65; 95% CI, 0.58–0.72; *P* < 0.001), in the sensitivity model with further adjustment using SAPS II and ICU interventions (HR, 0.75; 95% CI, 0.68–0.83; *P* < 0.001), and after replacing SAPS II with SOFA score while retaining adjustment for ICU interventions (HR, 0.75; 95% CI, 0.68–0.84; *P* < 0.001; Supplementary Table S8). E-value analysis was performed for the primary endpoint. For the association between PNI and 30-day mortality in the primary model, the E-value was 2.10 for the point estimate and 1.88 for the confidence limit closest to the null. In the sensitivity model, the corresponding E-values were 1.74 and 1.53, respectively.

Bootstrap-based resampling supported the stability of the primary findings (Supplementary Table S9). The bootstrap-based mean HRs for each 10-unit increase in PNI were 0.630 for 30-day mortality, 0.635 for 90-day mortality, and 0.683 for 365-day mortality, which were highly consistent with the original adjusted estimates.

In the independent eICU-CRD cohort, higher PNI remained significantly associated with lower in-hospital mortality (Supplementary Table S10). In the primary model, each 10-unit increase in PNI was associated with lower risk of in-hospital mortality (OR, 0.66; 95% CI, 0.57–0.76; *P* < 0.001). Compared with Group 1, the adjusted ORs were 0.62 (95% CI, 0.47–0.83) for Group 2, 0.47 (95% CI, 0.33–0.66) for Group 3, and 0.42 (95% CI, 0.29–0.62) for Group 4 (*P* for trend < 0.001).

## Discussion

In this retrospective cohort study of critically ill patients with AF, lower PNI was independently associated with higher risk of in-hospital, 30-day, 90-day, and 365-day mortality in the MIMIC-IV cohort. These associations remained robust across multiple sensitivity analyses, including additional adjustment for acute illness severity and early ICU interventions. A similar inverse association with in-hospital mortality was observed in the independent eICU-CRD cohort. In addition, Kaplan-Meier survival analysis demonstrated a clear graded relationship, with progressively worse survival observed in lower PNI categories. Furthermore, RCS analyses revealed a nonlinear inverse dose-response relationship between continuous PNI and mortality, indicating that the prognostic gradient is most evident at lower PNI levels.

In critically ill patients with AF, the arrhythmia is often not an isolated electrophysiological event but rather a manifestation of severe systemic stress characterized by inflammation, sympathetic activation, hemodynamic instability, tissue hypoperfusion, and multiorgan dysfunction [3, 8, 9]. Accordingly, adverse outcomes in this setting are unlikely to be driven by AF alone; rather, they probably reflect the interaction between the arrhythmia and the patient’s underlying biological resilience, cardiovascular reserve, and capacity to tolerate acute systemic stress [8, 9]. In this setting, the prognostic relevance of PNI likely reflects the clinical significance of its two components. Serum albumin is influenced not only by nutritional status but also by systemic inflammation, increased capillary permeability, hemodilution, and fluid resuscitation [11, 12, 25], whereas lymphocyte count may decrease in response to stress-related immune dysregulation, neurohormonal activation, and inflammatory burden [13, 26, 27]. Therefore, in critically ill patients, lower PNI should not be interpreted as evidence of malnutrition alone. Instead, it may reflect a broader state of clinical vulnerability shaped by inflammatory and nutritional disturbance, reduced physiologic reserve, and greater acute illness burden. This interpretation is consistent with the baseline profile observed in our cohort, in which lower PNI was associated with greater comorbidity burden, higher illness-severity scores, and more frequent use of organ-supportive therapies, suggesting a more vulnerable clinical phenotype at ICU admission.

A growing body of evidence has demonstrated the prognostic value of PNI across the spectrum of high-risk cardiovascular diseases. For instance, a recent comprehensive meta-analysis highlighted PNI as a robust predictor of adverse outcomes in patients with coronary artery disease [16]. Furthermore, in settings that closely mirror the extreme physiological stress of our cohort, PNI has been shown to independently predict all-cause mortality in critically ill patients presenting with acute myocardial infarction [17]. Beyond ischemic events, recent evidence from heart failure populations further elucidates the potential mechanisms underlying PNI’s prognostic value, suggesting it captures a state of multidimensional systemic vulnerability rather than isolated malnutrition. In elderly patients with chronic heart failure, PNI effectively stratifies long-term mortality risk, with its prognostic utility being strongly modulated by concurrent inflammatory markers such as hsCRP and suPAR [15]. Similarly, in the setting of acute heart failure, PNI critically modifies the ‘obesity paradox’; the survival advantage typically associated with a higher body mass index is substantially attenuated in patients with a low PNI [28]. This underscores that biological reserve, which integrates nutritional status and systemic inflammation, may be more relevant to survival than body mass alone. Extrapolating from this extensive cardiovascular literature to our ICU-AF cohort, the strong inverse association between PNI and mortality likely reflects a shared pathophysiological paradigm. In highly stressed cardiovascular environments, PNI may reflect diminished physiological reserve and heightened inflammatory and nutritional risk.

PNI also belongs to a broader class of composite risk scores increasingly used in cardiovascular disease. As an immuno-nutritional index derived from serum albumin and total lymphocyte count, PNI conceptually aligns with other multidimensional laboratory-based scores that integrate inflammatory, nutritional, or systemic vulnerability signals into a single measure. For example, the Naples Prognostic Score has been reported to predict long-term vascular outcomes such as saphenous vein graft disease after coronary artery bypass grafting [29], whereas inflammatory composite scores have shown prognostic value for in-hospital mortality in infective endocarditis [30]. In addition, inflammatory scores have been associated with left atrial thrombosis even in ischemic stroke patients without AF [31], which indirectly supports the notion that inflammation-related composite markers may reflect thromboinflammatory risk relevant to adverse cardiovascular outcomes. Similarly, the reported utility of the Intermountain Risk Score in predicting coronary artery ectasia [32] further suggests that composite scores may capture clinically relevant dimensions of risk not fully represented by conventional physiological severity measures. These studies collectively support that composite scores like PNI can capture multidimensional clinical vulnerability linked to adverse cardiovascular outcomes.

RCS analyses indicated a nonlinear association between PNI and mortality. A threshold effect was observed at a PNI value of approximately 40; below this level, each increase in PNI was associated with a markedly lower risk of 30-day mortality, whereas the association was attenuated above this level. This pattern suggests that the prognostic relevance of PNI may be greatest at lower values, where differences in biological reserve and inflammatory and nutritional status are likely to be more clinically apparent. In additional prognostic analyses, PNI provided only modest supplementary prognostic information beyond established ICU severity scores. Although reclassification indices improved after adding PNI to SAPS II or APACHE II, the absolute gains in C-statistics were small; similarly, adding PNI to SOFA resulted in only a minimal and non-significant change in the C-statistic. These findings indicate that PNI should not be interpreted as a replacement for established ICU severity scores or as a stand-alone prediction tool. Rather, PNI may serve as a simple adjunctive marker reflecting inflammatory, nutritional, and physiological vulnerability. From a clinical perspective, PNI offers several practical advantages: it can be calculated from routine laboratory tests obtained at ICU admission, requires no additional cost or specialized assays, and is readily available in time-sensitive or resource-limited settings. However, the present findings do not support interpreting PNI as a therapeutic target. Whether interventions aimed at improving nutritional status, modulating inflammation, or correcting lymphopenia would alter outcomes in this population cannot be inferred from the current observational data.

Several limitations should be acknowledged. First, this retrospective observational study cannot establish causal relationships. Residual confounding may exist because some potentially relevant factors—including frailty, sarcopenia, inflammatory biomarkers, congestion-related albumin dilution, and dynamic changes in albumin or lymphocyte count—were unavailable, and PNI was not compared with alternative nutritional indices. E-value analyses suggested that an unmeasured confounder of at least moderate strength would be required to fully explain the observed association.

Second, patients with liver disease or malignancy were excluded to reduce the influence of extreme pathological conditions on PNI, but this may have introduced selection bias, as the included cohort may not fully represent the broader population of critically ill patients with AF.

Third, reverse causality remains possible because lower PNI may partly reflect greater acute illness severity rather than pre-existing physiological vulnerability. However, the association persisted after adjustment for acute severity scores and ICU interventions and after excluding patients who died within 48 h of ICU admission.

Fourth, AF identification was based on ICD-9/10 diagnosis codes in MIMIC-IV and diagnosis text in eICU-CRD rather than systematic electrocardiographic adjudication. Previous validation studies have suggested that ICD-based algorithms can identify AF with acceptable accuracy in administrative and electronic health data; for example, a systematic review reported positive predictive values of 70%–96% for prevalent AF identified using ICD-9 code 427.31, with a median sensitivity of 79% across studies [33]. Nevertheless, coding-based and diagnosis-text-based definitions may still lead to misclassification, particularly regarding the timing of AF onset and the distinction between pre-existing AF and NOAF. Misclassification between pre-existing AF and NOAF remains possible because rhythm documentation may be incomplete and AF episodes occurring before ICU admission may not be fully captured.

Fifth, the independent eICU-CRD cohort analysis was limited to in-hospital mortality because long-term follow-up data were unavailable. Nevertheless, the replication of the inverse association between PNI and in-hospital mortality in an independent multicenter cohort supports the robustness of the main findings.

Finally, the prognostic association between PNI and mortality may not be unique to AF. Because this study did not include a non-AF ICU comparison group, we cannot determine whether the magnitude of this association or the identified risk threshold differs between patients with and without AF. Therefore, the findings should be interpreted as evidence supporting the prognostic relevance of PNI within a clinically important ICU-AF subgroup, rather than as evidence of AF-specific prognostic utility. Whether the risk thresholds identified in this specific high-risk population are generalizable to other critical illness cohorts requires further comparative evaluation.

## Conclusions

Lower PNI was independently associated with increased short- and long-term mortality in critically ill patients with AF. In this setting, a lower PNI may reflect broader clinical vulnerability beyond malnutrition, including systemic inflammation, reduced physiological reserve, and impaired tolerance to acute systemic stress. As a simple index derived from routine laboratory tests at ICU admission, PNI may provide adjunctive prognostic information in this high-risk population.

## Supplementary Information


Supplementary Material 1.



Supplementary Material 2.



Supplementary Material 3.



Supplementary Material 4.


## Data Availability

The datasets analyzed in the current study are available from PhysioNet. Specifically, this study used the Medical Information Mart for Intensive Care IV database (MIMIC-IV, version 3.1) and the eICU Collaborative Research Database (eICU-CRD, version 2.0). MIMIC-IV version 3.1 is available at https://physionet.org/content/mimiciv/3.1/, and eICU-CRD version 2.0 is available at https://physionet.org/content/eicu-crd/2.0/. Access to these databases is available to credentialed users who have completed the required training in human subjects research and signed the relevant data use agreements. Database access was obtained by Zongjun Hu after completion of the Collaborative Institutional Training Initiative (CITI) training (certification ID: 60604020). The code used for data extraction is available from the corresponding author upon reasonable request.

## Abbreviations

AF: Atrial fibrillation
PNI: Prognostic nutritional index
ICU: Intensive care unit
MIMIC-IV: Medical Information Mart for Intensive Care IV
eICU-CRD: eICU Collaborative Research Database
CKD: Chronic kidney disease
CHF: Congestive heart failure
CAD: Coronary artery disease
SBP: Systolic blood pressure
RRT: Renal replacement therapy
RCS: Restricted cubic splines
NOAF: New-onset atrial fibrillation
NRI: Net reclassification improvement
IDI: Integrated discrimination improvement
SAPS II: Simplified Acute Physiology Score II
APACHE II: Acute Physiology and Chronic Health Evaluation II
SOFA: Sequential Organ Failure Assessment
CCI: Charlson Comorbidity Index
HR: Hazard ratio
OR: Odds ratio
CI: Confidence interval
IQR: Interquartile range
VIF: Variance inflation factor
