# Supplementary material for: Association between prognostic nutritional index and mortality in critically ill patients with atrial fibrillation: a retrospective cohort study
Source: BMC Cardiovasc Disord. 2026 Jun 20;26:756. doi: 10.1186/s12872-026-06138-4 (PMC13528156; doi:10.1186/s12872-026-06138-4)
Supplement: Supplementary file 1 [file 12872_2026_6138_MOESM1_ESM.docx]

**Supplementary Tables: Association between prognostic nutritional index and mortality in critically ill patients with atrial fibrillation: a retrospective cohort study**

****Supplementary Table S1.**** Codes and diagnosis strings used to identify atrial fibrillation

### ****Supplementary Table S2.**** Medications administered within 24 h of ICU admission ****Supplementary Table S3.**** Variables with missing data in the MIMIC-IV and eICU-CRD cohorts ****Supplementary Table S4.**** Assessment of multicollinearity and confounding for covariate selection ****Supplementary Table S5.**** Baseline characteristics of participants according to PNI groups in the eICU-CRD cohort ****Supplementary Table S6.**** Comparison of discrimination metrics for 30-day mortality between PNI and its individual components ****Supplementary Table S7.**** Incremental prognostic value of PNI beyond SAPS II- and APACHE II-based models for 30-day mortality in the MIMIC-IV cohort ****Supplementary Table S8.**** Sensitivity analyses for the association between PNI (per 10-unit increase) and 30-day mortality in the MIMIC-IV cohort ****Supplementary Table S9.**** Bootstrap-based internal validation for the association between PNI (per 10-unit increase) and mortality in critically ill patients with atrial fibrillation

****Supplementary Table S10.**** Multivariable logistic regression analyses for the association between PNI and in-hospital mortality in the MIMIC-IV and eICU-CRD cohorts

**Supplementary Table S1.** Codes and diagnosis strings used to identify atrial fibrillation

| MIMIC-IV cohort | | |
| --- | --- | --- |
| Code system | Code | Description |
| ICD-9-CM | 42731 | Atrial fibrillation |
| ICD-10-CM | I48.0 | Paroxysmal atrial fibrillation |
| ICD-10-CM | I48.1 | Persistent atrial fibrillation |
| ICD-10-CM | I48.11 | Longstanding persistent atrial fibrillation |
| ICD-10-CM | I48.19 | Other persistent atrial fibrillation |
| ICD-10-CM | I48.2 | Chronic atrial fibrillation |
| ICD-10-CM | I48.20 | Chronic atrial fibrillation, unspecified |
| ICD-10-CM | I48.21 | Permanent atrial fibrillation |
| ICD-10-CM | I48.91 | Unspecified atrial fibrillation |
| eICU-CRD cohort | | |
| Field name | Diagnosis string | Description |
| Diagnosis string | cardiovascular\|arrhythmias\|atrial fibrillation | Atrial fibrillation |
| Diagnosis string | cardiovascular\|arrhythmias\|atrial fibrillation\|with rapid ventricular response | Atrial fibrillation with rapid ventricular response |
| Diagnosis string | cardiovascular\|arrhythmias\|atrial fibrillation\|with controlled ventricular response | Atrial fibrillation with controlled ventricular response |
| Diagnosis string | cardiovascular\|arrhythmias\|atrial fibrillation\|without hemodynamic compromise | Atrial fibrillation without hemodynamic compromise |
| Diagnosis string | cardiovascular\|arrhythmias\|atrial fibrillation\|with hemodynamic compromise | Atrial fibrillation with hemodynamic compromise |
| Diagnosis string | cardiovascular\|vascular disorders\|arterial thromboembolism\|embolic, atrial fibrillation | Embolic atrial fibrillation |
| Diagnosis string | general\|quality measures\|atrial fibrillation treated with anticoagulants | Atrial fibrillation treated with anticoagulants |
| Diagnosis string | surgery\|cardiac surgery\|arrhythmia\|atrial fibrillation\|with hemodynamic compromise | Atrial fibrillation with hemodynamic compromise after cardiac surgery |
| Diagnosis string | cardiovascular\|vascular disorders\|peripheral vascular ischemia\|embolic, atrial fibrillation | Embolic atrial fibrillation-related peripheral ischemia |
| Diagnosis string | surgery\|acute cardiac problems\|atrial fibrillation | Atrial fibrillation |
| Diagnosis string | surgery\|acute cardiac problems\|atrial fibrillation\|rapid ventricular response | Atrial fibrillation with rapid ventricular response |
| Diagnosis string | surgery\|cardiac surgery\|arrhythmia\|atrial fibrillation | Atrial fibrillation after cardiac surgery |
| Diagnosis string | surgery\|acute cardiac problems\|atrial fibrillation\|controlled ventricular response | Atrial fibrillation with controlled ventricular response |
| Diagnosis string | surgery\|acute cardiac problems\|atrial fibrillation\|with hemodynamic compromise | Atrial fibrillation with hemodynamic compromise |
| Diagnosis string | surgery\|acute cardiac problems\|atrial fibrillation\|without hemodynamic compromise | Atrial fibrillation without hemodynamic compromise |
| Diagnosis string | hematology\|coagulation disorders\|arterial thromboembolism\|embolic, atrial fibrillation | Embolic atrial fibrillation-related thromboembolism |
| Diagnosis string | surgery\|cardiac surgery\|arrhythmia\|atrial fibrillation\|without hemodynamic compromise | Atrial fibrillation without hemodynamic compromise after cardiac surgery |
| Diagnosis string | cardiovascular\|arrhythmias\|SVT\|Wolff-Parkinson-White syndrome\|with atrial fibrillation | Atrial fibrillation associated with Wolff-Parkinson-White syndrome |

****Abbreviations:**** ICD, International Classification of Diseases; MIMIC-IV, Medical Information Mart for Intensive Care IV; eICU-CRD, eICU Collaborative Research Database.

**Supplementary Table S2.** Medication categories and corresponding agents administered within the first 24 h of ICU admission

| Category | Medications |
| --- | --- |
| **Beta-blockers** | Metoprolol; Atenolol; Bisoprolol; Esmolol; Propranolol; Nadolol; Sotalol; Pindolol; Labetalol; Carvedilol |
| Anticoagulants | Heparin; Enoxaparin; Warfarin; Apixaban; Rivaroxaban; Dabigatran; Fondaparinux; Argatroban; Bivalirudin; Alteplase |
| **Diuretics** | Furosemide; Torsemide; Bumetanide; Ethacrynic acid; Hydrochlorothiazide; Chlorothiazide; Chlorthalidone; Metolazone; Indapamide; Spironolactone; Amiloride; Triamterene; Eplerenone; Acetazolamide; Mannitol |
| **Inotropic drugs** | Digoxin; Dopamine; Milrinone; Dobutamine; Epinephrine |
| **Vasopressors** | Vasopressin; Norepinephrine; Phenylephrine |

### **Supplementary Table S3.** Variables with missing data in the MIMIC-IV and eICU-CRD cohorts

| Variables | MIMIC-IV (*n* = 3007) |  | eICU-CRD (*n* = 2741) |  |
| --- | --- | --- | --- | --- |
|  | **Missing (n)** | **Missing (%)** | **Missing (n)** | **Missing (%)** |
| **Vital signs** |  |  |  |  |
| Temperature, °C | 9 | 0.30 | 76 | 2.77 |
| Respiratory rate, breaths/min | 3 | 0.10 | 10 | 0.36 |
| Systolic blood pressure, mmHg | 2 | 0.07 | 3 | 0.11 |
| Diastolic blood pressure, mmHg | 2 | 0.07 | 3 | 0.11 |
| **Laboratory tests** |  |  |  |  |
| Bicarbonate, mmol/L | 4 | 0.13 | 97 | 3.54 |
| Sodium, mmol/L | 4 | 0.13 | 2 | 0.07 |
| Potassium, mmol/L | 4 | 0.13 | 2 | 0.07 |
| Chloride, mmol/L | 4 | 0.13 | 2 | 0.07 |
| Platelet count, ×10⁹/L | 2 | 0.07 | 3 | 0.11 |
| Creatinine, mg/dL | 1 | 0.03 | 3 | 0.11 |

**Supplementary Table S4.** Assessment of multicollinearity and change-in-estimate for covariate selection

| Potential covariates | Change in PNI coefficient (%)^a^ | VIF | Selected |
| --- | --- | --- | --- |
| Age, years | 1.4 | 1.334 | Yes |
| Male, n (%) | 0.2 | 1.127 | Yes |
| Hypertension | 0.0 | 1.120 | Yes |
| Cerebrovascular disease | **12.6** | 1.202 | Yes |
| COPD | 0.6 | 1.092 | No |
| Diabetes | 0.0 | 1.146 | Yes |
| CKD | -1.4 | 1.448 | Yes |
| CAD | 0.0 | 1.149 | No |
| CHF | -0.5 | 1.369 | Yes |
| Valvular heart disease | -0.1 | 1.143 | No |
| SAPS II | **-49.8** | 1.761 | Yes |
| Temperature, °C | 0.8 | 1.167 | No |
| Heart rate, beats/min | -4.0 | 1.436 | Yes |
| Respiratory rate, breaths/min | -4.2 | 1.166 | Yes |
| SBP, mmHg | -2.8 | 1.645 | Yes |
| DBP, mmHg | -0.4 | 1.578 | No |
| SpO₂, % | -3.2 | 1.196 | No |
| Hemoglobin, g/dL | 7.3 | 1.386 | Yes |
| Platelet count, ×10⁹/L | 0.4 | 1.143 | No |
| Creatinine, mg/dL | -2.6 | 2.752 | Yes |
| Blood urea nitrogen, mg/dL | -7.9 | 2.345 | No |
| Bicarbonate, mmol/L | -2.3 | 1.872 | Yes |
| Sodium, mmol/L | 1.0 | **4.550** | No |
| Chloride, mmol/L | 0.8 | **4.808** | No |
| Potassium, mmol/L | 4.4 | 1.253 | No |
| Beta-blocker use | -2.5 | 1.171 | No |
| Anticoagulant use | -0.1 | 1.099 | No |
| Diuretic use | 1.0 | 1.252 | No |
| Vasopressor use | **-12.6** | 1.457 | Yes |
| Inotropic drug use | 0.4 | 1.150 | No |
| Mechanical ventilation | **-14.1** | 1.495 | Yes |
| RRT | -2.8 | 1.338 | Yes |

^a^ Change in the regression coefficient for PNI after addition of each covariate to the base model.Variables such as SAPS II and early ICU interventions were retained for the extended sensitivity model but not for the prespecified primary model because they may also act as proxies of severity or potential mediators.

****Abbreviations:**** PNI, prognostic nutritional index; COPD, chronic obstructive pulmonary disease; CKD, chronic kidney disease; CAD, coronary artery disease; CHF, congestive heart failure; SAPS II, Simplified Acute Physiology Score II; SBP, systolic blood pressure; DBP, diastolic blood pressure; SpO₂, peripheral oxygen saturation; RRT, renal replacement therapy; VIF, variance inflation factor.

**Supplementary Table S5.** Baseline characteristics according to PNI groups in the eICU-CRD cohort

| **Variables** | Total (n = 2741) | Group 1 (n = 998) | Group 2 (n = 654) | Group 3 (n = 556) | Group 4 (n = 533) | *P* value |
| --- | --- | --- | --- | --- | --- | --- |
| **Demographics** |  |  |  |  |  |  |
| Age, years | 76.0 (65.0–84.0) | 76.0 (67.0–84.0) | 77.0 (67.0–85.0) | 76.0 (65.0–84.0) | 71.0 (61.2–80.0) | < 0.001 |
| Male, n (%) | 1472 (53.7) | 545 (54.6) | 335 (51.2) | 306 (55.0) | 286 (53.7) | 0.504 |
| **Comorbidities,** n **(%)** |  |  |  |  |  |  |
| Hypertension | 1947 (71.0) | 694 (69.5) | 486 (74.3) | 399 (71.8) | 368 (69.0) | 0.129 |
| Cerebrovascular disease | 733 (26.7) | 246 (24.6) | 180 (27.5) | 158 (28.4) | 149 (28.0) | 0.304 |
| COPD | 744 (27.1) | 288 (28.9) | 178 (27.2) | 166 (29.9) | 112 (21.0) | 0.003 |
| Diabetes | 946 (34.5) | 349 (35.0) | 237 (36.2) | 200 (36.0) | 160 (30.0) | 0.100 |
| CKD | 616 (22.5) | 272 (27.3) | 157 (24.0) | 117 (21.0) | 70 (13.1) | < 0.001 |
| CAD | 970 (35.4) | 336 (33.7) | 238 (36.4) | 205 (36.9) | 191 (35.8) | 0.540 |
| CHF | 1057 (38.6) | 405 (40.6) | 274 (41.9) | 230 (41.4) | 148 (27.8) | < 0.001 |
| Valvular heart disease | 104 (3.8) | 50 (5.0) | 26 (4.0) | 17 (3.1) | 11 (2.1) | 0.026 |
| **Severity scores** |  |  |  |  |  |  |
| CCI | 5.0 (4.0–6.0) | 5.0 (4.0–7.0) | 5.0 (4.0–6.0) | 5.0 (4.0–6.0) | 4.0 (3.0–6.0) | < 0.001 |
| SAPS II | 32.0 (25.0–41.0) | 35.0 (28.0–45.0) | 32.0 (26.0–40.2) | 30.0 (24.0–39.0) | 27.0 (22.0–35.0) | < 0.001 |
| APACHE IV | 64.0 (50.0–80.0) | 73.0 (58.0–90.0) | 64.0 (50.0–78.0) | 59.0 (48.0–74.0) | 53.0 (41.0–67.0) | < 0.001 |
| **Vital signs** |  |  |  |  |  |  |
| Temperature, °C | 36.7 (36.4–37.1) | 36.8 (36.4–37.2) | 36.7 (36.4–37.0) | 36.7 (36.4–37.0) | 36.7 (36.4–36.9) | < 0.001 |
| Heart rate, beats/min | 98.0 (81.0–117.0) | 100.0 (84.0–119.0) | 97.0 (79.0–115.8) | 97.0 (78.0–116.0) | 97.0 (80.0–116.0) | 0.001 |
| Respiratory rate, breaths/min | 21.0 (18.0–25.0) | 21.0 (18.0–26.0) | 21.0 (18.0–25.0) | 20.0 (17.0–25.0) | 21.0 (17.0–25.0) | 0.068 |
| SBP, mmHg | 119.0 (102.0–140.0) | 112.0 (96.0–130.0) | 119.0 (103.0–140.8) | 126.0 (107.0–149.0) | 125.0 (110.0–145.0) | < 0.001 |
| DBP, mmHg | 67.0 (56.0–81.0) | 62.0 (52.0–74.0) | 67.0 (56.0–81.0) | 72.0 (60.8–87.0) | 74.0 (63.0–88.0) | < 0.001 |
| SpO₂, % | 97.0 (94.0–99.0) | 97.0 (94.0–100.0) | 97.0 (94.0–99.0) | 97.0 (94.5–99.0) | 97.0 (95.0–99.0) | 0.358 |
| **Laboratory tests** |  |  |  |  |  |  |
| WBC, ×10⁹/L | 11.0 (8.0–15.2) | 12.0 (8.3–17.2) | 10.9 (7.9–14.9) | 10.3 (7.4–14.1) | 10.4 (8.3–14.2) | < 0.001 |
| Hemoglobin, g/dL | 11.9 (10.1–13.6) | 10.5 (9.1–12.1) | 11.7 (10.0–13.3) | 12.8 (11.2–14.2) | 13.6 (12.3–15.0) | < 0.001 |
| Platelet count, ×10⁹/L | 205.0 (158.0–265.0) | 199.0 (142.0–269.0) | 201.0 (157.0–263.8) | 199.0 (165.5–257.5) | 222.0 (180.5–269.0) | < 0.001 |
| Creatinine, mg/dL | 1.2 (0.9–1.9) | 1.3 (0.9–2.2) | 1.2 (0.9–2.0) | 1.2 (0.9–1.7) | 1.1 (0.8–1.4) | < 0.001 |
| Blood urea nitrogen, mg/dL | 26.0 (17.0–42.0) | 31.0 (20.0–49.0) | 26.0 (17.0–45.0) | 24.0 (17.0–37.0) | 20.0 (15.0–28.0) | < 0.001 |
| Bicarbonate, mmol/L | 24.0 (21.0–27.0) | 24.0 (20.2–27.4) | 25.0 (21.2–28.0) | 24.2 (22.0–28.0) | 24.0 (22.0–27.0) | 0.080 |
| Sodium, mmol/L | 138.0 (135.0–141.0) | 138.0 (135.0–141.0) | 138.0 (134.0–141.0) | 138.0 (135.0–141.0) | 138.0 (136.0–141.0) | 0.022 |
| Chloride, mmol/L | 103.0 (98.0–106.0) | 103.0 (99.0–108.0) | 102.0 (98.0–106.0) | 102.0 (97.8–106.0) | 102.0 (99.0–105.0) | < 0.001 |
| Potassium, mmol/L | 4.1 (3.7–4.6) | 4.1 (3.7–4.6) | 4.1 (3.7–4.6) | 4.2 (3.8–4.7) | 4.0 (3.7–4.4) | < 0.001 |
| **Treatments,** n **(%)** |  |  |  |  |  |  |
| Beta-blocker use | 690 (25.2) | 193 (19.3) | 165 (25.2) | 157 (28.2) | 175 (32.8) | < 0.001 |
| Anticoagulant use | 818 (29.8) | 258 (25.9) | 190 (29.1) | 184 (33.1) | 186 (34.9) | < 0.001 |
| Diuretic use | 498 (18.2) | 127 (12.7) | 131 (20.0) | 146 (26.3) | 94 (17.6) | < 0.001 |
| Vasopressor use | 424 (15.5) | 239 (23.9) | 86 (13.1) | 56 (10.1) | 43 (8.1) | < 0.001 |
| Inotropic drug use | 207 (7.6) | 87 (8.7) | 39 (6.0) | 46 (8.3) | 35 (6.6) | 0.141 |
| Mechanical ventilation | 631 (23.0) | 294 (29.5) | 126 (19.3) | 118 (21.2) | 93 (17.4) | < 0.001 |
| RRT | 108 (3.9) | 54 (5.4) | 29 (4.4) | 18 (3.2) | 7 (1.3) | < 0.001 |
| **Outcomes,** n **(%)** |  |  |  |  |  |  |
| In-hospital mortality | 416 (15.2) | 221 (22.1) | 92 (14.1) | 58 (10.4) | 45 (8.4) | < 0.001 |

Data are presented as **median (interquartile range [IQR])** or n (%), as appropriate. P values were calculated using the Kruskal-Wallis test for continuous variables and the chi-square test or Fisher’s exact test, as appropriate, for categorical variables. PNI groups were defined based on the quartiles of the MIMIC-IV cohort: Group 1, PNI < 33.95; Group 2, 33.95 ≤ PNI < 39.80; Group 3, 39.80 ≤ PNI < 45.25; and Group 4, PNI ≥ 45.25.

****Abbreviations:**** PNI, prognostic nutritional index; COPD, chronic obstructive pulmonary disease; CKD, chronic kidney disease; CAD, coronary artery disease; CHF, congestive heart failure; CCI, Charlson Comorbidity Index; SAPS II, Simplified Acute Physiology Score II; APACHE IV, Acute Physiology and Chronic Health Evaluation IV; SBP, systolic blood pressure; DBP, diastolic blood pressure; SpO₂, peripheral oxygen saturation; WBC, white blood cell count; RRT, renal replacement therapy; **IQR, interquartile range.**

**Supplementary Table S6.** Comparison of discrimination metrics for 30-day mortality between PNI and its individual components

| Comparator | C-statistic (95% CI) | *P* value | Continuous NRI (95% CI) | *P* value | IDI (95% CI) | *P* value |
| --- | --- | --- | --- | --- | --- | --- |
| **PNI** | **0.612 (0.588–0.636)** | **Ref** |  | **Ref** |  | Ref |
| Lymphocyte count | 0.591 (0.567–0.615) | 0.080 | 0.115 (0.056–0.170) | < 0.001 | 0.019 (0.010–0.031) | < 0.001 |
| Albumin | 0.591 (0.566–0.616) | < 0.001 | 0.120 (0.042–0.165) | 0.02 | 0.007 (0.002–0.013) | 0.008 |

****Abbreviations:**** PNI, prognostic nutritional index; CI, confidence interval; NRI, net reclassification improvement; IDI, integrated discrimination improvement.

**Supplementary Table S7.** Incremental prognostic value of PNI beyond SAPS II- and APACHE II-based models for predicting 30-day mortality in the MIMIC-IV cohort

| Model | C-statistic (95% CI) | *P* value | Continuous NRI (95% CI) | *P* value | IDI (95% CI) | *P* value |
| --- | --- | --- | --- | --- | --- | --- |
| SAPS II | 0.711 (0.690–0.732) | Ref |  | Ref |  | Ref |
| SAPS II + PNI | 0.717 (0.696–0.738) | 0.094 | 0.095 (0.052–0.138) | < 0.001 | 0.007 (0.002–0.017) | < 0.001 |
| APACHE II | 0.687 (0.665–0.709) | Ref |  | Ref |  | Ref |
| APACHE II + PNI | 0.698 (0.676–0.720) | 0.015 | 0.105 (0.060–0.153) | < 0.001 | 0.009 (0.003–0.020) | < 0.001 |

****Abbreviations:**** PNI, prognostic nutritional index; SAPS II, Simplified Acute Physiology Score II; APACHE II, Acute Physiology and Chronic Health Evaluation II; CI, confidence interval; NRI, net reclassification improvement; IDI, integrated discrimination improvement; MIMIC-IV, Medical Information Mart for Intensive Care IV.

****Supplementary Table S8.**** Sensitivity analyses for the association between PNI (per 10-unit increase) and 30-day mortality in the MIMIC-IV cohort

| Category | Sensitivity Analysis Scenarios | n | Adjusted HR (95% CI) | P value |
| --- | --- | --- | --- | --- |
| **I. Population exclusion** | 1. Exclusion of patients with CHF | 1,432 | 0.69 (0.60–0.80)^a^ | < 0.001 |
|  | 2. Exclusion of patients with CAD | 1,702 | 0.60 (0.52–0.69)^a^ | < 0.001 |
|  | 3. Exclusion of patients with valvular heart disease | 2,346 | 0.63 (0.56–0.71)^a^ | < 0.001 |
|  | 4. Exclusion of early deaths (≤ 48h) | 2,890 | 0.60 (0.54–0.68)^a^ | < 0.001 |
| **II. Robustness of adjustment** | 5. Further adjustment for overall comorbidity burden (CCI) | 3,007 | 0.65 (0.58–0.72)^b^ | < 0.001 |
|  | 6. Additional adjustment for acute severity and ICU interventions | 3,007 | 0.75 (0.68–0.83)^c^ | < 0.001 |

**Notes:**
a. **Standard adjustment (baseline for Scenarios 1-4):** Adjusted for age, sex, hypertension, cerebrovascular disease, diabetes, CKD, CHF (except in Scenario 1), heart rate, respiratory rate, SBP, hemoglobin, creatinine, and bicarbonate.
b. **Comorbidity adjustment:** Adjusted for age, sex, hypertension, heart rate, respiratory rate, SBP, hemoglobin, creatinine, bicarbonate, and **CCI**. To avoid multicollinearity, individual comorbidities (CHF, CAD, CKD, diabetes, and cerebrovascular disease) were replaced by CCI score in this model.
c. **Maximum Adjustment:** Adjusted for all variables in Footnote (a) plus **SAPS II score, vasopressor use, mechanical ventilation, and renal replacement therapy (RRT).** This scenario corresponds to Model 3 in Table 2.

****Abbreviations:**** PNI, prognostic nutritional index; HR, hazard ratio; CI, confidence interval; CHF, congestive heart failure; CAD, coronary artery disease; CKD, chronic kidney disease; CCI, Charlson Comorbidity Index; SBP, systolic blood pressure; SAPS II, Simplified Acute Physiology Score II; RRT, renal replacement therapy.

**Supplementary Table S9.** Bootstrap-based internal validation of the association between PNI (per 10-unit increase) and mortality in critically ill patients with atrial fibrillation

| Endpoint | Original adjusted HR | Bootstrap mean HR | Bootstrap median HR | Bootstrap percentile-based 95% CI |
| --- | --- | --- | --- | --- |
| 30-day mortality | 0.631 | 0.630 | 0.631 | 0.560–0.705 |
| 90-day mortality | 0.636 | 0.635 | 0.636 | 0.576–0.701 |
| 365-day mortality | 0.685 | 0.683 | 0.682 | 0.626–0.751 |

Bootstrap resampling was performed with 1,000 repetitions based on the primary adjusted Cox regression model (Model 2), which was adjusted for **age, sex, hypertension, cerebrovascular disease, diabetes, CKD, CHF, heart rate, respiratory rate, SBP, hemoglobin, creatinine, and bicarbonate**. The bootstrap 95% confidence interval was estimated using the percentile method.

**Abbreviations:** HR, hazard ratio; CI, confidence interval; PNI, prognostic nutritional index.

**Supplementary Table S10.** Multivariable logistic regression analyses of the association of PNI with in-hospital mortality in the MIMIC-IV and eICU-CRD cohorts

| **PNI** | **Crude model** | |  | **Model 1** | |  | **Model 2** | |  | **Model 3** | |
| --- | --- | --- | --- | --- | --- | --- | --- | --- | --- | --- | --- |
|  | OR **(95%** CI**)** | ***P* value** |  | OR **(95%** CI**)** | ***P* value** |  | OR **(95%** CI**)** | ***P* value** |  | OR **(95%** CI**)** | ***P* value** |
| **MIMIC-IV cohort** |  |  |  |  |  |  |  |  |  |  |  |
| Per 10-unit increase | 0.60 (0.53–0.67) | < 0.001 |  | 0.60 (0.53–0.67) | < 0.001 |  | 0.58 (0.50–0.66) | < 0.001 |  | 0.71 (0.61–0.81) | < 0.001 |
| Q1 | 1.00 (Ref) |  |  | 1.00 (Ref) |  |  | 1.00 (Ref) |  |  | 1.00 (Ref) |  |
| Q2 | 0.54 (0.43–0.69) | < 0.001 |  | 0.53 (0.41–0.67) | < 0.001 |  | 0.55 (0.42–0.70) | < 0.001 |  | 0.61 (0.46–0.79) | < 0.001 |
| Q3 | 0.41 (0.32–0.53) | < 0.001 |  | 0.40 (0.31–0.52) | < 0.001 |  | 0.41 (0.31–0.54) | < 0.001 |  | 0.57 (0.42–0.76) | < 0.001 |
| Q4 | 0.40 (0.31–0.52) | < 0.001 |  | 0.41 (0.32–0.53) | < 0.001 |  | 0.40 (0.29–0.53) | < 0.001 |  | 0.58 (0.43–0.80) | 0.001 |
| *P* **for trend** |  | **< 0.001** |  |  | **< 0.001** |  |  | **< 0.001** |  |  | **< 0.001** |
| **eICU-CRD cohort** |  |  |  |  |  |  |  |  |  |  |  |
| Per 10-unit increase | 0.60 (0.53–0.68) | < 0.001 |  | 0.62 (0.54–0.70) | < 0.001 |  | 0.66 (0.57–0.76) | < 0.001 |  | 0.75 (0.64–0.88) | < 0.001 |
| Group 1 | 1.00 (Ref) |  |  | 1.00 (Ref) |  |  | 1.00 (Ref) |  |  | 1.00 (Ref) |  |
| Group 2 | 0.58 (0.44–0.75) | < 0.001 |  | 0.60 (0.46–0.78) | < 0.001 |  | 0.62 (0.47–0.83) | 0.001 |  | 0.75 (0.55–1.02) | 0.071 |
| Group 3 | 0.41 (0.30–0.56) | < 0.001 |  | 0.42 (0.30–0.57) | < 0.001 |  | 0.47 (0.33–0.66) | < 0.001 |  | 0.53 (0.36–0.77) | 0.001 |
| Group 4 | 0.32 (0.23–0.46) | < 0.001 |  | 0.36 (0.25–0.51) | < 0.001 |  | 0.42 (0.29–0.62) | < 0.001 |  | 0.56 (0.37–0.85) | 0.007 |
| P **for trend** |  | **< 0.001** |  |  | **< 0.001** |  |  | **< 0.001** |  |  | **< 0.001** |

Participants were stratified into four groups (Group 1 to Group 4) based on the quartiles of PNI in the MIMIC-IV cohort. For external validation, the same thresholds were applied to the eICU-CRD cohort to ensure consistency in clinical stratification. The specific cutoff values were: Group 1, PNI < 33.95; Group 2, 33.95 ≤ PNI < 39.80; Group 3, 39.80 ≤ PNI < 45.25; and Group 4, PNI ≥ 45.25.
The crude model was unadjusted.
**Model 1** was adjusted for age and sex.
**Model 2** was adjusted for age, sex, hypertension, cerebrovascular disease, diabetes, CKD, CHF, heart rate, respiratory rate, SBP, hemoglobin, creatinine, and bicarbonate.
**Model 3** was adjusted for age, sex, hypertension, cerebrovascular disease, diabetes, CKD, CHF, heart rate, respiratory rate, SBP, hemoglobin, creatinine, and bicarbonate, SAPS II, vasopressor use, mechanical ventilation, and RRT.

****Abbreviations:**** PNI, prognostic nutritional index; MIMIC-IV, Medical Information Mart for Intensive Care IV; eICU-CRD, eICU Collaborative Research Database; OR, odds ratio; CI, confidence interval; CKD, chronic kidney disease; CHF, congestive heart failure; SBP, systolic blood pressure; SAPS II, Simplified Acute Physiology Score II; RRT, renal replacement therapy.
