# Supplementary material for: Association between prognostic nutritional index and mortality in critically ill patients with atrial fibrillation: a retrospective cohort study
Source: BMC Cardiovasc Disord. 2026 Jun 20;26:756. doi: 10.1186/s12872-026-06138-4 (PMC13528156; doi:10.1186/s12872-026-06138-4)
Supplement: Supplementary file 2 [file 12872_2026_6138_MOESM2_ESM.docx]

**Supplementary Fig. 1. Schoenfeld residual plots assessing the proportional hazards assumption for PNI.**
Schoenfeld residual plots for the association between PNI and mortality over time. Panel A shows the analysis for 30-day mortality, Panel B for 90-day mortality, and Panel C for 365-day mortality. The horizontal reference line indicates the null effect, the dashed cyan line represents the average hazard, and the solid red line represents the time-varying hazard. A generally stable fitted curve around the reference line suggests no obvious violation of the proportional hazards assumption for PNI across the three mortality endpoints.
