# Supplementary material for: Association between prognostic nutritional index and mortality in critically ill patients with atrial fibrillation: a retrospective cohort study
Source: BMC Cardiovasc Disord. 2026 Jun 20;26:756. doi: 10.1186/s12872-026-06138-4 (PMC13528156; doi:10.1186/s12872-026-06138-4)
Supplement: Supplementary file 3 [file 12872_2026_6138_MOESM3_ESM.docx]

### Supplementary Table S11. Tests of the proportional hazards assumption for PNI using scaled Schoenfeld residuals

| **Mortality endpoint** | **Variable tested** | **Chi-square** | **df** | **P value** | **Interpretation** |
| --- | --- | --- | --- | --- | --- |
| 30-day mortality | PNI | 3.166 | 1 | 0.075 | No significant violation |
| 90-day mortality | PNI | 2.465 | 1 | 0.116 | No significant violation |
| 365-day mortality | PNI | 0.172 | 1 | 0.678 | No significant violation |

**Abbreviations:** PNI, prognostic nutritional index; df, degrees of freedom.
**Note:** The proportional hazards assumption for PNI was assessed using scaled Schoenfeld residuals in the adjusted Cox models. A two-sided P value < 0.05 was considered evidence of a statistically significant violation of the proportional hazards assumption.
